# Evolutionary and taxonomic insights into the genomic divergence of cowpea mild mottle virus: rapid evolution in replication-associated protein gene, but strong negative selection on coat protein gene

**DOI:** 10.1099/jgv.0.002262

**Published:** 2026-05-28

**Authors:** Bruno Arcanjo Silva, João Marcos Fagundes Silva, Caterynne Melo Kauffmann, Stephanny Barreto dos Santos Cárdenas, Paloma de Souza Queiroz, Amanda Moraes do Vale Batista, Helena Beatriz da Silva Mota, Rosana Blawid, Débora Maria Sansini Freitas, Alice Kazuko Inoue-Nagata, Tatsuya Nagata

**Affiliations:** 1 Departamento de Fitopatologia, Universidade de Brasília, Brasília, Distrito Federal, Brazil; 2 Instituto de Ciências Biológicas, Universidade de Brasília, Brasília, Distrito Federal, Brazil; 3 Departamento de Agronomia, Universidade Federal Rural de Pernambuco, Recife, Pernambuco, Brazil; 4 Embrapa Semiárido, Petrolina, Pernambuco, Brazil; 5 Embrapa Hortaliças, Samambaia Norte, Distrito Federal, Brazil

**Keywords:** *Betaflexiviridae*, carlavirus, virus evolution, virus taxonomy

## Abstract

*Carlavirus vignae*, with the common name cowpea mild mottle virus (CPMMV), has become a widespread carlavirus infecting economically important crops worldwide. CPMMV is transmitted by whiteflies, whereas other members of the *Carlavirus* genus are generally transmitted by aphids. Recently, the virus has been isolated from various host plants, including cucurbit plants in Brazil, exhibiting a wide genomic variation. Here, we present a comprehensive analysis of CPMMV genome diversity, focusing on the replication-associated protein (Rep) and coat protein (CP) genes, two key genomic regions for *Betaflexiviridae* classification. Cucurbit isolates of CPMMV from Brazil showed high divergent Rep but low CP aa sequences among them. Based on pairwise aa sequence identity of the Rep, the CPMMV available in public databases can be divided into at least four distinct genogroups. In contrast, CP sequences exhibited a remarkable sequence conservation across all analysed isolates, likely due to strong negative selection maintaining its functional or structural integrity. Furthermore, we concluded that the recombination is not the reason for the higher conservation of the CP relative to the Rep. These findings support the hypothesis that, in the family *Betaflexiviridae*, the Rep evolves at a more constant rate, whereas the CP evolves by ‘leaps’ of fast evolution. Since the differences in Rep aa sequence identities are well below the threshold defined by the *Betaflexiviridae* species demarcation criteria, we propose classifying the CPMMV isolates into four distinct species.

## Data Availability

The genome sequences determined in this study have been deposited in GenBank with the following accession numbers: LC877455, LC877456 and LC877457. The other datasets generated during and/or analysed as part of this study are available from the corresponding author on reasonable request.

## Introduction

Carlaviruses are positive-sense ssRNA viruses belonging to the family *Betaflexiviridae* (order *Tymovirales*). They have monopartite, polyadenylated RNA genomes and the virion is filamentous and flexuous. The genome encodes a replication-associated protein (Rep) that contains the conserved methyltransferase (Met), helicase (Hel) and RNA-dependent RNA polymerase (RdRp) domains [1]. The family *Betaflexiviridae* currently includes 15 genera, grouped into 2 subfamilies: *Quinvirinae*, for which the genome encodes a triple gene block (TGB) involved in intercellular virus movement [2,3], and *Trivirinae*, which encodes a 30K-like movement protein for cell-to-cell transport [4]. The genus *Carlavirus* is, thus, part of the subfamily *Quinvirinae*. Transmission of carlaviruses typically occurs via aphid vectors; however, two carlaviruses, cowpea mild mottle virus (CPMMV, *Carlavirus vignae*) and melon yellowing-associated virus (MYaV, *Carlavirus melonis*), are transmitted by whiteflies (*Bemisia tabaci*) [5,6].

CPMMV was first reported in 1973 in Ghana infecting cowpea (*Vigna unguiculata*) plants [7]. Since then, various CPMMV isolates have been reported worldwide, primarily infecting fabaceous hosts. However, CPMMV was also found in plants from other botanical families, including eggplant (*Solanum melongena*) in Jordan [8], chia (*Salvia hispanica*) in Argentina [9], passionfruit (*Passiflora* spp.) in Brazil [10] and hibiscus (*Hibiscus syriacus*) in the Netherlands [11]. More recently, we detected another CPMMV isolate, which was infecting cucurbit species, such as melon (*Cucumis melo*) and cucumber (*Cucumis sativus*), and inducing yellowing symptoms in Brazil [12]. In addition, we detected two genetically distinct CPMMV variants from melon in the present study. These reports suggest that CPMMV either has a broad host range or it has adapted to infect non-fabaceous plants. Here, we analysed the full genome sequences of all CPMMV isolates, those sequenced in this study and retrieved from public databases, and observed a high degree of genetic variability, supporting the hypothesis that CPMMV is rapidly evolving and may constitute a species complex. The possibility of reclassifying CPMMV into at least four distinct viral species is discussed.

## Methods

### Material collection, sample preparation and genome sequencing of CPMMV

Leaf samples with viral symptoms such as mosaic and yellowing from plants in the *Cucumis* genus, including melon, cucumber and West Indian gherkin (*Cucumis anguria*), in the commercial fields in the Sinop region, Mato Grosso State, Brazil, were collected in September 2022. The virome study of these samples was performed using a semi-purified viral preparation from pooled leaf samples, as described by Silva *et al*. [12]. Briefly, total RNA was extracted from this preparation and subjected to high-throughput sequencing (HTS) on the Illumina NovaSeq platform (150 bp paired-end reads, 10 G scale) at Macrogen Inc. (Seoul, South Korea). HTS reads were trimmed and assembled into contigs using Megahit v1.2.9 [13]. The resulting contigs were analysed using blastx against the viral genome database in Geneious v8.1.9 (Biomatters, Auckland, New Zealand). In the first round of the analysis of these HTS-derived reads, one CPMMV genotype was identified in the single melon plant (QAU20933), named as Melon_1 isolate and reported [12]. However, a more in-depth analysis of the HTS reads of the same cohorts revealed two additional putative genotypes of CPMMV, designated Melon_2 and Melon_3. The complete genomes of both genotypes were first assembled by mapping all HTS-derived reads, followed by re-sequencing the genomic cDNA prepared from a single plant for each genotype using the Sanger method. For this purpose, total RNA was extracted from individual melon leaf samples stored at −80 °C. Reverse transcription-polymerase chain reaction (RT-PCR) was performed using genotype-specific detection primers for Melon_1, Melon_2 and Melon_3 (Table S1, available in the online Supplementary Material) to identify the individual samples containing each of the three genotypes. To sequence the complete genomes of Melon_2 and Melon_3 genotypes, reverse transcription was performed using Superscript IV (Thermo Fisher Scientific, Waltham, USA), and the genomic cDNA fragments were amplified using LongAmp *Taq* DNA polymerase (New England Biolabs, Ipswich, USA) across three overlapping regions of ~3 kbp each using genotype-specific primers (Table S1). The 5′-RACE [14] and 3′-RACE [15] protocols were conducted to determine the 5′ and 3′ genomic termini using specific primers (Table S1). All cDNA amplicons were directly sequenced by the Sanger method with primer walking.

The fourth CPMMV isolate was obtained from a common bean (*Phaseolus vulgaris*) plant collected in Santo Antônio de Goiás, Brazil (Bean-1 isolate) and sequenced for comparison using the same RT-PCR sequencing procedure described above.

### Taxonomic and phylogenetic analysis

The three new CPMMV sequences (Melon-2, Melon-3 and Bean-1) from this study and 55 complete genome sequences retrieved from GenBank were used for Rep and CP (aa sequence comparison). Pairwise aa sequence identities among CPMMV isolates were calculated for the Rep and CP regions using MAFFT v7.520 [16] and custom scripts. Phylogenetic trees were inferred based on the aa sequences of the Rep and CP using IQ-TREE2 v2.3.6 [17], with the best-fit substitution models selected by ModelFinder [18]. Branch support was inferred by the SH-aLRT and ultrafast bootstrap methods, with 1,000 replicates each.

### Analysis of suppression of synonymous site variability

The suppression of synonymous mutation variability along the concatenated coding sequences of Rep and CP was analysed using SynPlot2 [19]. The coding sequences were aligned using the codon-msa script from the HyPhy package [20] (https://github.com/veg/hyphy-analyses/tree/master/codon-msa) and aligned using MAFFT v7.520 [15].

### Recombination analysis

The complete genome nt sequences of 58 CPMMV isolates available in databases were aligned using muscle [21]. Recombination analysis was then performed with RDP5 software [22], employing all available methods except LARD (likelihood analysis of recombination in DNA). Only recombination events supported by at least four distinct methods were considered for further evaluation. The distance plot was made using RDP5 with Melon_1 isolate as the reference. To investigate recombination events with CPMMV and other carlaviruses, another RDP5 analysis was conducted with the same parameters. Due to mutation saturation, alignments at the nt level become less reliable as the distance between sequences increases. Thus, in order to detect recombination events between CPMMV and other species without compromising the detection of recombination within CPMMV, a second analysis was made with an alignment containing the 58 CPMMV isolates and the representative sequence of all other carlavirus species.

### Selection analysis

The coding sequences of the Rep and CP were aligned using the codon-msa script of the HyPhy package [20] (https://github.com/veg/hyphy-analyses/tree/master/codon-msa). Prior to the selection analysis, Rep and CP alignments were partitioned based on recombination breakpoints identified using GARD [23]. Selection pressures were subsequently estimated with BUSTED [24] and FEL [25].

## Results

### CPMMV melon isolates in Brazil are divergent

The complete genomes of three new isolates of CPMMV, two from melon (Melon_2 and Melon_3, accession numbers LC877455 and LC877456, respectively) and one from common bean (Bean_1, LC877457), were sequenced in this study. They were sequenced directly from PCR amplicons from individual samples, from which each CPMMV variant was detected using specific primers (Table S1). The genomes were 8,194 to 8,196 nt long and shared >98% nt identity with other reported CPMMV isolates, except for Melon-2.

Then, the analysis focused on Rep and CP aa sequences, the most relevant part of the genome for taxonomy. The Rep aa sequence identities among the melon isolates, including Melon_1 (previously reported, PV448278), were significantly low, ranging from 78.3 to 79.6% (mean=78.87, sd=0.65; Table 1). This level is slightly lower than the threshold of 80% aa sequence identity for distinct virus species. However, CP aa identities among them exceeded 98% (mean=98.96, sd=0.35; Table 1). These findings suggest that, according to the demarcation criteria proposed by [26], they belong to the same species, despite their significant divergence. When the sequence of Bean_1 was included in the comparison, it showed 98.0% aa identity in Rep and 99.3% in CP with Melon_1. This implied that host species might not be a major factor for CPMMV evolution.

**Table 1.** Amino acid sequence identities of Rep and CP among melon isolates of CPMMV

CP\Rep	**Melon_1**	**Melon_2**	**Melon_3**
**Melon_1**		**78.87**	**78.33**
**Melon_2**	**98.96**		**79.62**
**Melon_3**	**98.61**	**98.26**	

### CPMMV sequences are grouped into at least four distinct genogroups based on Rep aa comparison

**Fig. 1.**
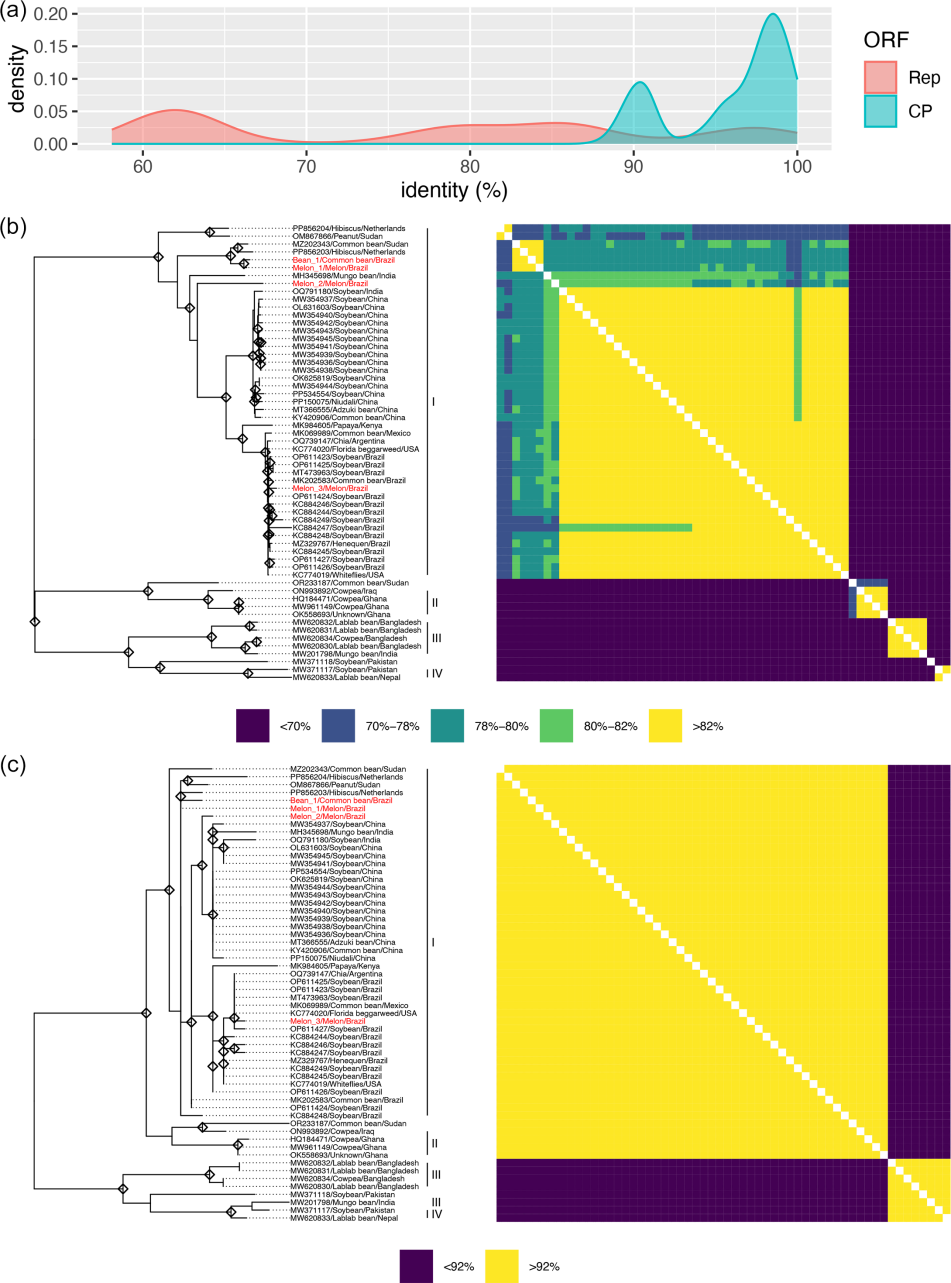
Taxonomic and phylogenetic analysis of CPMMV isolates. (**a**) Distribution of pairwise aa identity values for the Rep and CP sequences, represented as kernel density plots. (**b**) Maximum likelihood phylogenetic tree and pairwise identity heatmap for Rep. Colour coding in the heatmap reflects thresholds used for species demarcation, with the addition of the 70% threshold that was chosen based on the distribution observed in panel (**a**). (**c**) Maximum likelihood phylogenetic tree and pairwise identity heatmap for CP. Since pairwise identities exceed 85%, 92% aa identity threshold was applied for colour scaling, based on the distribution valley observed in panel (**a**), to enhance visualization. The novel Brazilian isolates are shown in red in both trees, and diamonds represent branches with bootstrap support >70%.

To further elucidate the taxonomic and evolutionary relationships among CPMMV isolates, all full genome sequences available in databases were retrieved (*n*=58) and pairwise aa identities were calculated for the Rep and CP (Fig. 1a), followed by phylogenetic inference (Fig. 1b, c). The percent identities among each pairwise combinations were counted, transformed into density values and plotted in a graph (Fig. 1a). Two completely distinct peaks were recognized for Rep and for CP (Fig. 1a). A considerable part of the pairwise identities was below 70% for the Rep protein. For classification in one species in the *Carlavirus* genus, these identity values are far below the threshold of 80% of the species demarcation criteria, indicating these viruses can be potentially classified as distinct species. In contrast, for the CP analysis, the first peak was observed ~90% and the second at 98%. The CP aa identity is the second parameter for species demarcation criteria with a threshold of 85%, when the Rep protein identity shows values in the 78–82% borderline [26]. Therefore, the value is still within the range of the same species. Based on Rep aa identities, CPMMV sequences can be divided into at least four distinct genogroups (I to IV in Fig. 1b). The largest genogroup, Genogroup I with 45 sequences, primarily comprised isolates from the Americas and Asia, especially from Brazil and China, and included all Brazilian melon isolates. Genogroup II consisted of four isolates from Ghana and Iraq (Africa and the Middle East). The third genogroup (III) was formed with five isolates from Bangladesh and India (South Asia) and the fourth genogroup (IV) with two isolates from Nepal and Pakistan (South Asia). In contrast, the CP sequences displayed a high degree of conservation at the aa level (Fig. 1a, c). Isolates of Genogroups I and II shared higher than 92% identity, as well as those of groups III and IV (South Asian). Two isolates, OR233187 and MW371118, despite having Rep pairwise identities to other isolates below 78 and 70%, respectively, were not grouped in genogroups since each would be composed of only one isolate (Fig. 1b), and thus, more sequences are necessary to establish them within distinct genogroups.

In Genogroup I, 37 isolates (mainly from Brazil and China) share Rep aa identities >82%, indicating they are properly classified within one species. Another group of six isolates, including Melon_2, share identities from 78 to 82%, i.e. in the boundary of species demarcation. However, two isolates, from the Netherlands and Sudan, share 70–78% identities. This indicated that within Genogroup I, further splitting might be applicable considering the species demarcation threshold.

**Fig. 2.**
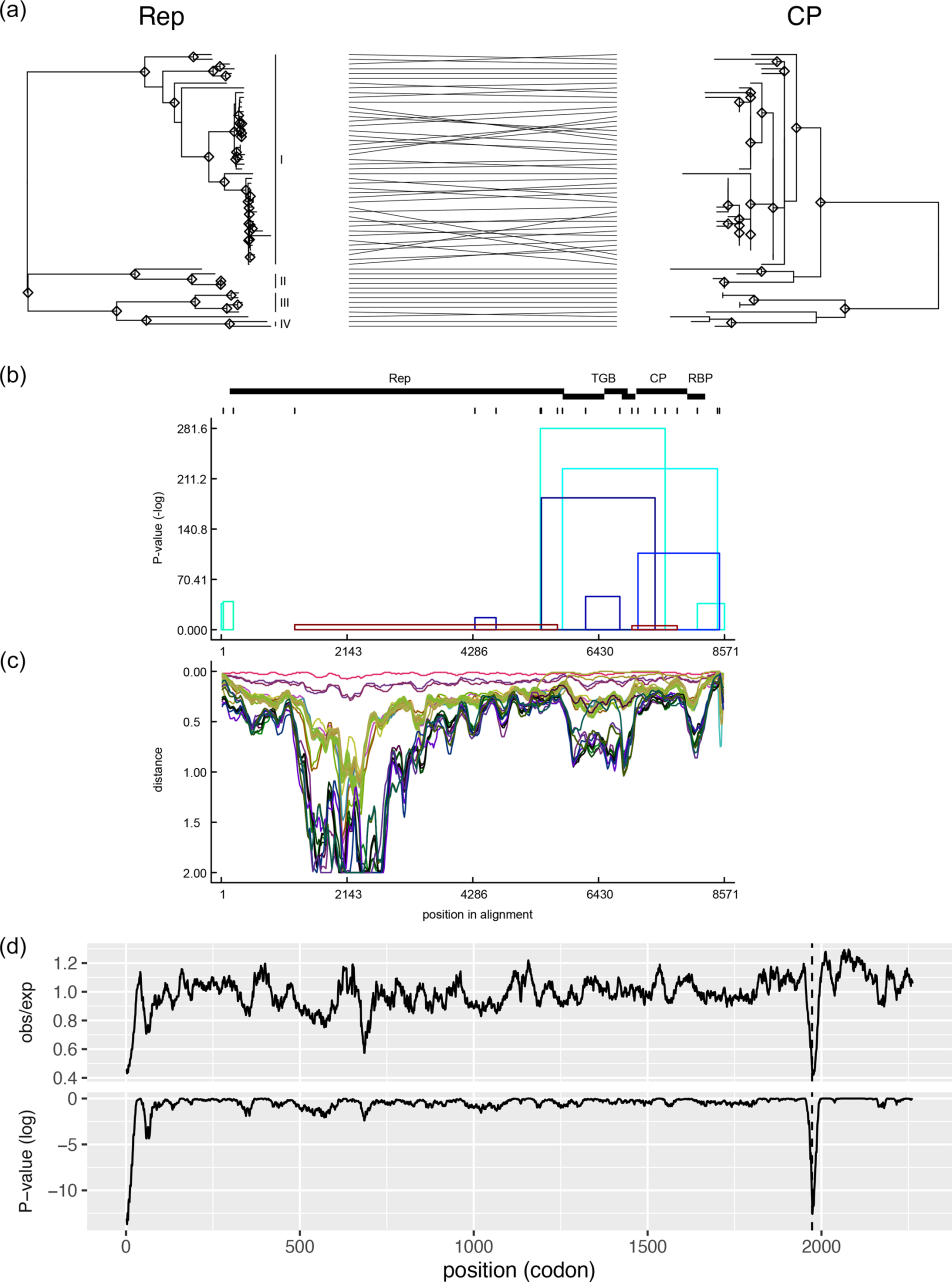
Evolution of the Rep and CP. (**a**) Tanglegram showing the position of an isolate in both the Rep and CP phylogenies. Diamonds represent branches with bootstrap support >70%. (**b**) Recombination event map, with recombination breakpoints shown above the plot as vertical bars and ORFs. Rectangular bars (upper) showed the positions of each gene. Rep, replication-associated protein; TGB, triple gene block; CP, coat protein; RBP, RNA-binding protein. Asterisks indicate recombination events found at the Rep. (**c**) Pairwise distance plot using isolate Melon_1 as a representative sequence. (**d**) Synonymous sites variability suppression per codon (top) and the associated *P*-value (bottom). The junction point between the Rep and CP sequences is indicated by a dashed line.

Recombination events directly influence pairwise identities of the genomes. For instance, in a species composed of two genogroups, a recombination event between their ancestral isolates reduces genomic pairwise identities if the ancestral sequences are lost. In this scenario, for some portions of the genome, only one of the genogroups is found, reducing pairwise identities. Alternatively, if some isolates within one species recombine with another species, pairwise identities increase. Accordingly, low diversity in the CP of CPMMV in comparison to the Rep sequences can be explained by recombination with the further loss of the parental sequences. Therefore, to investigate the evolutionary trajectories of the Rep and CP genes, we constructed a tanglegram comparing their phylogenetic trees (Fig. 2a). From this analysis, the putative recombination can be deduced by the crossing lines between Rep and CP phylogenies. Most recombination events occurred between isolates from the same continent mainly in Genogroup I, suggesting a pattern of geographically restricted recombination. Notably, no substantial evidence of recombination between different genogroups was found (except one suspected case between Genogroup III – MW201798/Mungo bean/India and Genogroup IV – MW371118/Soybean/Pakistan, both are of South Asian groups). Additional recombination analysis performed with RDP5 supported these observations. Moreover, recombination breakpoints, especially those with high statistical support, were located closer to the 3′ region of the genome (Fig. 2b).

In addition to the above hypothesis, recombination events in the Rep with other species could in turn increase the pairwise identities of the Rep, especially if breakpoints are located within the Rep. Accordingly, pairwise distances of Melon_1 isolate against other viruses in the CPMMV species complex (Fig. 2c) show a region of increased diversity in the Rep. Nevertheless, only two recombination events were found at the Rep, albeit with low statistical support (Fig. 2b, indicated by asterisks). Whereas one event spanned past this high diversity region, the other is located entirely away from it. Additionally, a second recombination analysis with all 58 CPMMV isolates and 1 representative sequence from each species in the genus *Carlavirus* detected only 1 recombination event between CPMMV and an unknown species (positions 9 to 158 in Melon_1). This small event was found in several CPMMV isolates and may be an alignment artefact. Hence, there is no evidence that recombination is the cause of this increased variability among genogroups. Moreover, based on the distance plot, we can observe that the more C-terminal region of Rep (RNA-dependent RNA polymerase domain) and CP were more conserved than other regions of the CPMMV genome, such as the N-terminal region of Rep, TGBs and RNA-binding protein genes (Fig. 2c).

Although no recent recombination events were detected among distinct genogroups, we cannot exclude the possibility of an ancient recombination event involving ancestral lineages, which could have contributed to the level of conservation in the CP coding region. Since synonymous mutations are expected to accumulate at a relatively constant rate compared to nonsynonymous mutations, we employed synonymous mutation accumulation as a molecular clock to determine whether the CP is evolutionarily more recent than the Rep. If the CP accumulates less synonymous mutations than expected, in comparison to the Rep, then this would indicate that the CP is indeed more recent, supporting the hypothesis of a recombination event involving the ancestral sequences of the tentative novel species. However, our analysis of concatenated Rep and CP coding sequences revealed no evidence of suppressed synonymous site variability in the CP (Fig. 2d). The short regions exhibiting synonymous site variability are likely functional secondary structures, except for the region at the junction of the Rep and CP sequences, which is most likely an artefact. These results indicate that recombination is unlikely to be the main factor responsible for the higher conservation of the CP among these genogroups.

To further investigate the evolutionary constraints acting on the Rep and CP, we conducted a selection analysis to determine whether it is subject to stronger negative selection than the Rep. Prior to this analysis, recombination tests were performed using GARD. The inferred trees for each partition showed similar results to RDP5, where most viruses from the same genogroup remained together in all trees, indicating that recombination mostly took place within genogroups (Fig. S1). Subsequently, gene-wide episodic selection detection was conducted with BUSTED, utilizing three partitions for the Rep and two for the CP. Episodic positive selection was detected in the Rep (*P*=0), but not in the CP (*P*=0.06), supporting the hypothesis that stronger negative selection on the CP contributes to its conservation. Additionally, selection analysis using FEL revealed that 76.3% of invariant sites in the Rep and 93.4% in the CP are under negative selection (*P*<0.05). Furthermore, a single site under positive selection (*P*<0.05) was identified in the Rep, located at position 691 in the alignment (position 633 in the reference CPMMV from Ghana; accession: HQ184471, RefSeq: NC_014730).

## Discussion

The findings from this study provide compelling evidence that CPMMV comprises at least four distinct genogroups, as determined by the aa sequence identities of the Rep using all complete CPMMV genome sequences available in public databases (GenBank, EMBL and DDBJ). The newest demarcation criteria for the members of *Betaflexiviridae* is 80% in Rep aa sequence identity. The four genogroups shown in our study have much less Rep aa sequence identities below 70%, it is enough different to be classified as distinct species; i.e. the so-called CPMMV can be divided into four distinct species. While the CP gene is generally conserved across isolates, its lower pairwise aa variability compared to the Rep suggests that distinct evolutionary pressures are acting on this protein. The almost absence of recombination events among the genogroups detected in our analysis further suggests that factors beyond genetic exchange are driving the observed conservation of the CP. A plausible explanation for this pattern is differential selective pressures acting on the Rep and CP. Our selection analysis revealed significant evidence of episodic positive selection acting on Rep (*P*=0), while CP exhibited no significant signs of positive selection (*P*=0.06). These findings imply that Rep may undergo adaptive evolution, possibly in response to host immune pressures or functional constraints inherent to viral replication. In contrast, CP appears to be under stronger purifying selection, preserving its functions and structure that are essential for vector transmission, host interactions and virion stability [27,33]. The high conservation of CP across diverse CPMMV isolates aligns with trends observed in other viruses [28,31], where structural proteins tend to exhibit slower evolutionary rates compared to replication-associated proteins [27,32]. This pattern has also been reported in other *Carlavirus* species, suggesting that the conservation of the CP is likely linked to its role in vector specificity and host adaptation [26,34,36].

In general, plant host and vector adaptations are key factors in rapid viral evolution. Genogroup I comprises CPMMV isolates from diverse hosts, remarkably spanning at least six botanical families: *Fabaceae*, *Malvaceae*, *Cucurbitaceae*, *Caricaceae*, *Lamiaceae* and *Asparagaceae*. In contrast, other genogroups are restricted to fabaceous hosts. It is likely that fewer studies have focused on Genogroups II–IV, which have predominantly been reported from Asian countries. Consequently, host adaptation is difficult to evaluate as a determinant in CPMMV evolutionary processes.

Although comparing CPMMV’s evolutionary patterns with those of other whitefly-transmitted carlaviruses (WTCs) would be interesting, such analyses are limited because the only other WTC, MYaV, has just a single complete genome sequence available in databases.

In summary, these results indicate that differential selective pressures, rather than recombination, have been the primary drivers of the contrasting evolutionary patterns of Rep and CP in CPMMV. While Rep undergoes episodic adaptive changes, CP remains highly conserved, underscoring its pivotal role in the virus life cycle. Future studies incorporating additional CPMMV isolates and functional assays will be necessary to further elucidate the evolutionary dynamics shaping CPMMV diversity and adaptation.

## Supplementary material

10.1099/jgv.0.002262 Uncited Fig. S1.

10.1099/jgv.0.002262 Uncited Table S1.

## Abbreviations

CP: coat protein
CPMMV: cowpea mild mottle virus
HTS: high-throughput sequencing
MYaV: melon yellowing-associated virus
Rep: replication-associated protein
TGB: triple gene block
