# Supplementary material for: Evolutionary and taxonomic insights into the genomic divergence of cowpea mild mottle virus: rapid evolution in replication-associated protein gene, but strong negative selection on coat protein gene
Source: J Gen Virol. 2026 May 28;107(5):002262. doi: 10.1099/jgv.0.002262 (PMC13218543; doi:10.1099/jgv.0.002262)
Supplement: Uncited Fig. S1. [file jgv-107-02262-s001.pdf]

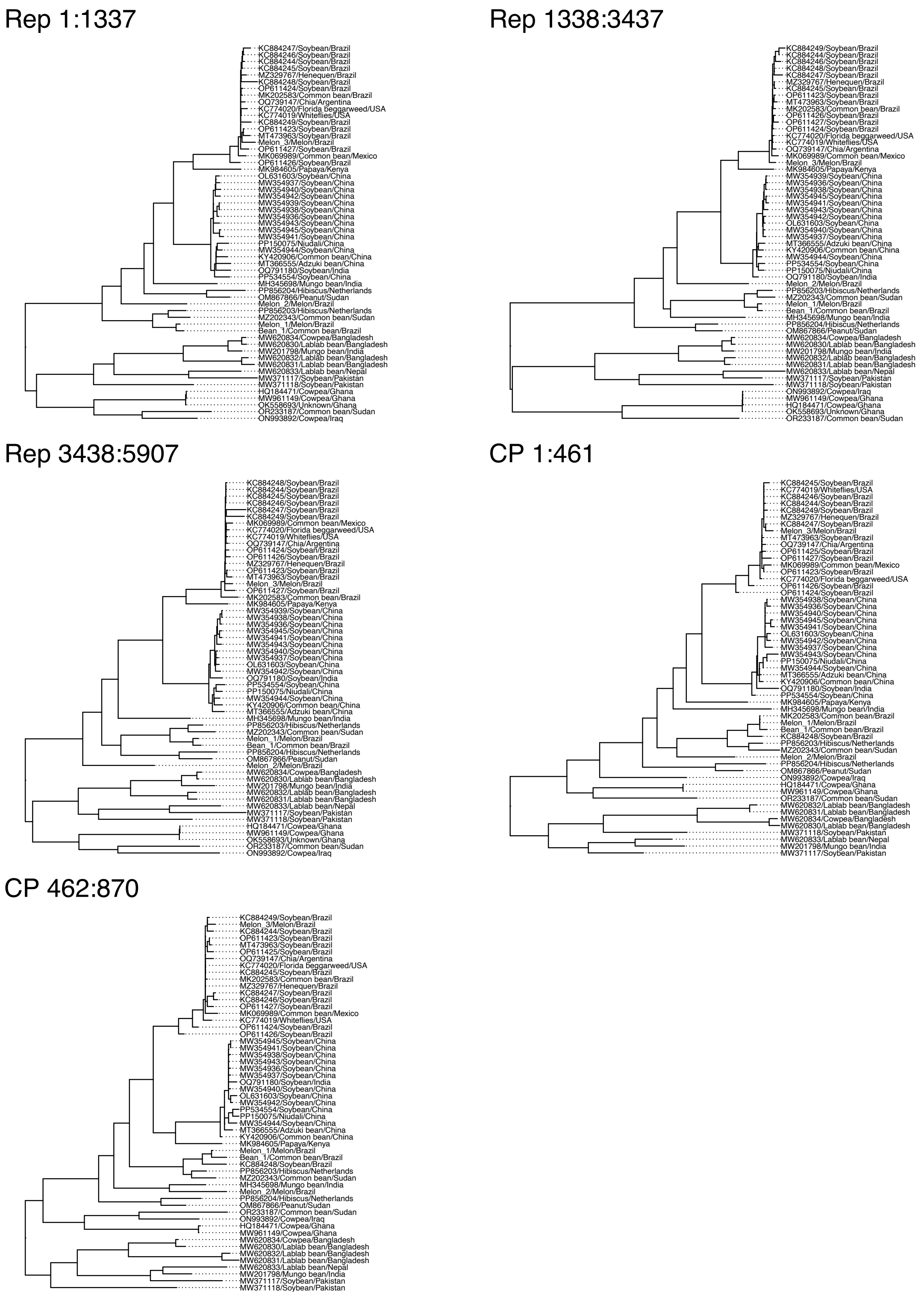

Figure S1. Phylogenetic trees of each recombinant partition obtained with GARD. Trees are midpoint-rooted for better visualization. The start and end position of each partition is shown at the title of each tree.
